# Supplementary material for: Associations between Suboptimal Sleep and Smoking, Poor Nutrition, Harmful Alcohol Consumption and Inadequate Physical Activity (‘SNAP Risks’): A Comparison of People with and without a Mental Health Condition in an Australian Community Survey
Source: Int J Environ Res Public Health. 2021 Jun 1;18(11):5946. doi: 10.3390/ijerph18115946 (PMC8199510; doi:10.3390/ijerph18115946)
Supplement: Supplementary file 1 [file ijerph-18-05946-s001.zip › ijerph-1223734-supplementary.pdf]

Supplementary Table 1. NSF Criteria for Categorising Sleep Parameters as 'Appropriate', 'May be appropriate' and 'Suboptimal', According to Age.

Sleep parameter	Age	Appropriate	May be appropriate	Suboptimal
**Sleep duration (hours)**	18-25	7-9	6, 10-11	< 6, >11
	26-64	7-9	6, 10	< 6, >10
	65+	7-8	5-6, 9	<5, >9
**Sleep onset latency (minutes)**	18-25	≤30	>30 ≤45	>45
	26-64	≤30	>30 ≤45	>45
	65+	≤30	>30 ≤60	> 60
**Awakenings (>5min)**	18-25	≤1	>1 ≤3	>3
	26-64	≤1	>1 ≤3	>3
	65+	≤2	>2 ≤3	>3
**Wake after sleep onset (minutes)**	18-25	≤20	>20 ≤40	>40
	26-64	≤20	>20 ≤40	>40
	65+	≤30	>30	N/A
**Sleep efficiency (%)**	18-25	≥85	<85 ≥65	<65
	26-64	≥85	<85 ≥75	<75
	65+	≥85	<85 ≥75	<75
**Naps per day (n)**	18-25	0	1-2	>2
	26-64	N/A	0-3	>3
	65+	N/A	0-3	>3
**Nap duration (minutes)**	18-25	N/A	0-100	>100
	26-64	N/A	0-100	>100
	65+	N/A	0-100	>100
**Nap frequency (days)**	18-25	0	≥1	N/A
	26-64	N/A	N/A	N/A
	65+	N/A	N/A	N/A

N/A: not applicable, no 'suboptimal' criteria.

Supplementary Table 2. Variations in Associations between Suboptimal Sleep and SNAP risks According to Mental Health Status

Mental Health Status	SNAP risk	OR	95 % CI		
			Lower	Upper	
Sleep Duration (Weekdays)[1]					
MHC[2]	Smoking risk	2.10	0.80	5.48	
	No smoking risk	2.33	1.22	4.44	
MHC[2]	Nutrition risk	2.33	1.22	4.44	
	No nutrition risk	1.89	0.54	6.62	
MHC[2]	Alcohol risk	3.06	1.41	6.64	*
	No alcohol risk	1.60	0.73	3.49	
MHC[2]	PA risk	1.89	0.54	6.62	
	No PA risk	1.89	0.54	6.62	
Sleep Duration (Weekends)[1]					
MHC[2]	Smoking risk	1.87	0.71	4.90	
	No smoking risk	2.62	1.37	5.03	
MHC[2]	Nutrition risk	2.49	1.61	3.84	
	No nutrition risk	1.97	0.56	6.97	
MHC[2]	Alcohol risk	3.06	1.41	6.69	*
	No alcohol risk	1.60	0.73	3.52	
MHC[2]	PA risk	2.20	1.01	4.80	
	No PA risk	2.23	1.02	4.88	
Sleep Onset Latency [1]					
MHC[2]	Smoking risk	3.37	1.01	11.24	
	No smoking risk	1.85	0.75	4.56	
MHC[2]	Nutrition risk	2.79	1.72	4.54	
	No nutrition risk	2.24	0.39	12.95	
MHC[2]	Alcohol risk	1.87	0.65	5.34	
	No alcohol risk	3.34	1.21	9.20	
MHC[2]	PA risk	2.27	0.80	6.46	
	No PA risk	2.75	1.01	7.45	
Awakenings[1]					
MHC[2]	Smoking risk	4.12	1.31	12.93	
	No smoking risk	3.17	1.48	6.80	
MHC[2]	Nutrition risk	2.44	1.41	4.21	
	No nutrition risk	5.37	1.27	22.66	
MHC[2]	Alcohol risk	3.13	1.23	7.97	
	No alcohol risk	4.17	1.66	10.49	
MHC[2]	PA risk	3.42	1.36	8.66	
	No PA risk	3.82	1.52	9.54	

Wake After Sleep Onset[1]					
MHC[2]	Smoking risk	5.07	1.12	22.95	
	No smoking risk	2.58	0.93	7.18	
MHC[2]	Nutrition risk	2.04	1.04	4.02	
	No nutrition risk	6.41	0.88	46.55	
MHC[2]	Alcohol risk	2.58	0.75	8.82	
	No alcohol risk	5.08	1.49	17.25	
MHC[2]	PA risk	3.14	0.93	10.61	
	No PA risk	4.16	1.23	14.05	
Sleep Efficiency[1]					
MHC[2]	Smoking risk	3.26	0.60	17.66	
	No smoking risk	1.86	0.55	6.22	
MHC[2]	Nutrition risk	2.83	1.38	5.79	
	No nutrition risk	2.14	0.20	22.84	
MHC[2]	Alcohol risk	1.98	0.49	8.04	
	No alcohol risk	3.06	0.74	12.63	
MHC[2]	PA risk	3.38	0.86	13.28	
	No PA risk	1.79	0.42	7.59	
Nap Duration[1]					
MHC[2]	Smoking risk	2.64	0.51	13.65	
	No smoking risk	2.69	0.70	10.36	
MHC[2]	Nutrition risk	2.25	1.01	5.02	
	No nutrition risk	3.16	0.27	37.14	
MHC[2]	Alcohol risk	4.70	1.06	20.93	
	No alcohol risk	1.51	0.34	6.68	
MHC[2]	PA risk	7.96	1.90	33.22	*
	No PA risk	0.89	0.17	4.57	

MHC: People with a mental health condition; PA: Physical activity

[1] Reference category: Appropriate/may be appropriate; [2] Reference category: No mental health condition

*Parameter estimate for interaction term significant at 0.05
